# A Hole Nucleation Method Combining BESO and Topological Sensitivity for Level Set Topology Optimization

**DOI:** 10.3390/ma14092119

**Published:** 2021-04-22

**Authors:** Shuangyuan Cao, Hanbin Wang, Jianbin Tong, Zhongqi Sheng

**Affiliations:** Institute of Advanced Manufacturing and Automation Technology, College of Mechanical Engineering and Automation, Northeastern University, Shenyang 110819, China; c1638182063@163.com (S.C.); 18740003357@163.com (H.W.); evantjb@163.com (J.T.)

**Keywords:** BESO, hole nucleation, level set method, topological sensitivity, topology optimization

## Abstract

As is known to all, the incapacity to nucleate holes automatically in the design domain is one of the main issues of the classical level set topology optimization method. To solve the issue of hole nucleation, this paper employs the bi-directional evolutionary structural optimization (BESO) method based on the material removal scheme and the frequently used topological sensitivity and proposes the combining BESO and topological sensitivity (CBT) method for level set topology optimization. This method can replace the existing hole nucleation method for level set topology optimization. First, the topological sensitivity is combined with BESO, and the BESO method based on topological sensitivity is proposed. Second, the method is integrated into level set topology optimization to solve the issue of hole nucleation. Two sensitivity thresholds are defined depending on the evolutionary volume ratio and boundary topological sensitivity, respectively, and the smaller one is used as the sensitivity threshold for hole nucleation. The material is removed from the design domain to nucleate holes based on this threshold. Three classical two-dimensional numerical examples are used to validate the proposed hole nucleation method.

## 1. Introduction

The topology optimization method can achieve the optimal distribution of materials in a specific area according to the given design domain, boundary conditions and loads. This method, which is not restricted by the experience of designers, can obtain novel high-quality structural configurations. It is convenient for designers to explore structures with better performance. It has been proven to be a powerful tool for optimal material distribution during the part design process and has a wide range of application prospects. There are many methods proposed for structural topology optimization, such as the homogenization method [1,2,3,4,5], solid isotropic material with penalization (SIMP) method [6,7,8,9], (bi-directional) evolutionary structural optimization method [10,11,12,13,14], moving morphable components (MMC) method [15,16,17,18], level set method [19,20,21,22,23,24,25], etc. In particular, the level set method can naturally perform boundary merging and movement. The level set method was first proposed by Osher and Sethian [26] to track and simulate the evolution of the dynamic boundary. Since it can implicitly describe the topological boundary, the level set method is applied to the field of structural topology optimization. The level set topology optimization method embeds the n-dimensional structural boundary into the (*n* + 1)-dimensional level set function, and the updated structural boundary is obtained via the evolution of the level set function. The form of implicit representation can avoid the slack of design variables and have high computational accuracy. Furthermore, the level set function can implicitly describe boundaries, which avoids numerical instability. These characteristics make the level set topology optimization method research focus on the field of structural optimization.

In the classical level set topology optimization method, the level set function is defined by a signed distance function. Since the level set function is reinitialized to maintain the signed distance function during the optimization process, the holes cannot be nucleated automatically in the design domain [20,27]. The level set topology optimization method employing the Hamilton–Jacobi equation as the boundary evolutionary equation, so it can be categorized as a shape optimization method. In other words, the topological changes of the level set topology optimization method can only be realized by the existing topology splitting and merging. Therefore, most of the classical level set topology optimization iterates from the initial structure with many holes, which makes the optimization result rely on the distribution of the initial holes. However, it is not easy to find an appropriate initial structure. At present, researchers have proposed related solutions to the issue. In particular, the method of nucleating holes using topological sensitivity is used widely. Allaire et al. [28] periodically compared the topological sensitivity values during the level set optimization process to insert holes in the design domain. However, this method is not easy to program and easily leads to accidental hole nucleation. Challis [21] and He et al. [29] added topological sensitivity to the Hamilton–Jacobi equation as a diffusive term. In this method, the premise that holes can be naturally nucleated during structural evolution is an appropriate parameter. In addition, Xia et al. [30] proposed a bi-directional evolutionary structural optimization (BESO) method based on the material removal scheme which automatically inserts holes in the optimization process. The concept of this evolution-based method is simple but lacks theoretical support. Yaghmaei et al. [27] proposed a filtering-based level set method, which can overcome the nucleation of holes and reinitialization of the level set function. However, the above methods based on the Hamilton–Jacobi evolution equation require a reasonable selection of parameters to make the optimization results converge stably. Furthermore, there are some level set variant functions that can achieve the effect of automatically nucleating holes. For example, the parameterized level set method (PLSM) based on radial basis functions (RBFs) proposed by Wang et al. [31,32,33] does not need to initialize the structure and can automatically nucleate holes during the optimization process. Compared with the method based on the Hamilton–Jacobi evolution equation, however, the computational cost of PLSM is expensive.

The above methods are only effective for hole nucleation under suitable parameters or specific conditions. In the present paper, the combining BESO and topological sensitivity (CBT) method is proposed as an effective alternative method for hole nucleation. This method combines the BESO with the concept of topological sensitivity frequently used in hole nucleation. Then the CBT method is integrated into the level set topology optimization method to realize the automatic nucleation of holes during the optimization process.

BESO is developed from evolutionary structural optimization (ESO). ESO was originally proposed by Xie and Steven [13] in 1992. This method is based on a simple algorithm, i.e., gradually removing low-efficiency materials in the structure to evolve the structure into an optimal result. Although this method can theoretically obtain the optimal solution, it will produce numerical problems such as checkerboard and mesh dependency. The main reason is that the ESO method can only remove low-efficiency materials but cannot add high-efficiency materials so that some materials with higher utilization are removed accidentally. In response to the above problems, Xie et al. [14] proposed the BESO that can remove low-efficiency materials and add materials simultaneously at key positions. This method is widely used in the engineering field because of its simple algorithm and easy programming.

In this paper, topological sensitivity is used as an important parameter of the hole nucleation method. The idea of topological sensitivity was first proposed by Eschenauer et al. [34], and then Sokolowski and Zochowoski [35] gave the definition of topological sensitivity and proposed a method for solving topological sensitivity based on shape sensitivity. Based on their topological sensitivity calculation method, Novotny et al. [36,37] calculated the topological sensitivity of two- and three-dimensional linear elastic problems. Otomori [22] obtained the topological sensitivity of the minimum compliance problem of the three-dimensional elastic continuum. The emergence of these efforts makes the application of topological sensitivity being further promoted.

BESO removes low-efficiency materials or adds high-efficiency materials to optimize the structure according to a certain criterion. Topological sensitivity describes the impact of optimization objective as inserting holes in the design domain. Therefore, using topological sensitivity as the criterion for adding or removing materials in the BESO accords with physical meaning. In this paper, topological sensitivity is used as the criterion for adding or removing materials in the BESO method and integrated into the classical level set topology optimization method to solve the problem that the classical level set method cannot automatically insert holes. This method fully combines the characteristics of stable material removal of BESO and easy calculation of topological sensitivity. Furthermore, the algorithm has the advantages of simple logic and easy programming and can be easily applied to various fields.

The rest of this paper is organized as follows. Section 2 describes the level set topology optimization method. Section 3 reviews the BESO method and the topological sensitivity. Section 4 proposes an improved BESO method based on topological sensitivity. Section 5 introduces the optimization problem, i.e., the CBT level set topology optimization. In Section 6, three numerical examples are used to analyze and demonstrate the method proposed in this paper. Finally, this paper is summarized in Section 7.

## 2. Level Set Topology Optimization Method

In the continuum structure level set topology optimization, an optimal design Ω is sought in the design domain *D*, and ∂Ω is the boundary of Ω. The topology of the structure is described by the implicit level set function *Φ*(***x***) as:

(1) $$\{\begin{array}{l} \Phi \left(x\right)>0\;\;\;x\in \Omega \;\;\;\;\;\;\;\;\;\\ \Phi \left(x\right)=0\;\;\;x\in \partial \Omega \;\;\;\;\;\;\;\\ \Phi \left(x\right)<0\;\;\;x\in D\cap \bar{\Omega } \end{array}$$

where ***x*** is an arbitrary point in the design domain *D*. Introducing the virtual time *t*, the Hamilton–Jacobi partial differential equation used to update the level set function is expressed as:

(2) $$\frac{\partial \Phi }{\partial t}-V_{n}\left|\nabla \Phi \right|=0$$

where *V_n_* is the evolutionary velocity of the boundary. The equation can be solved by the upwind finite difference scheme. Given the characteristics of the level set function, re-initialization needs to be performed periodically during the optimization.

Employing the level set function *Φ*(***x***), the level set topology optimization problem is written as follows:

(3) $$\begin{array}{c} min\;J(u,\Phi )=\int_{\Omega }f(u)H(\Phi )dx=\int_{\Omega }E\epsilon (u)\cdot \epsilon (v)H(\Phi )dx\\ s.t.\;a(u,v,\Phi )=L(v,\Phi )\;\;\;\;\;\\ \forall v\in U\;u|\Gamma_{D}=u_{0}\;\;\;\;\;\;\;\\ V(\Phi )\leq V_{M}\;\;\;\;\;\;\;\; \end{array}$$

where *u* is the displacement field, *E* is the elasticity tensor of the material, ϵ is the strain tensor, *P* is the body force, *τ* is the boundary force, $\Gamma_{D}$ is Dirichlet boundary conditions, *V_M_* is the maximum admissible volume, a(u,v,Φ), L(v,Φ) and V(Φ) are expressed as:

(4) $$\begin{array}{l} a(u,v,\Phi )=\int_{\Omega }E\epsilon (u)\cdot \epsilon (v)H(\Phi )dx\\ L(v,\Phi )=\int_{\Omega }PvH(\Phi )dx+\int_{\partial \Omega }\tau v\delta (\Phi )|\nabla \Phi |ds\\ v(\Phi )=\int_{\Omega }H(\Phi )dx \end{array}$$

where *H* (⋅) is the Heaviside function, which is defined as:

(5) $$H\left(x\right)=\{\begin{array}{c} 1\;\;x\geq 0\\ 0\;\;x<0 \end{array}$$

δ (⋅) is the Dirichlet function, and its relationship with the Heaviside function is as follows:

(6) $$\delta \left(x\right)=\frac{dH\left(x\right)}{dx}$$

To solve the Hamilton–Jacobi partial differential equation, the concept of shape sensitivity is introduced to calculate the velocity field *V_n_* of the level set function. The Murat and Simon analysis [38] based on the Hadamard variational method was used to calculate shape sensitivity. Considering a smooth initial shape Ω_0_, all admissible shapes Ω are obtained by applying a smooth vector field *θ*:

(7) $$\Omega =\left\{x+\theta \left(x\right),x\epsilon \Omega_{0}\right\}$$

The above equation shows that all admissible shapes are represented by a vector θ, so Equation (7) is also written as:

(8) $$\Omega =\left(\mathrm{Id}+\theta \right)\left(\Omega_{0}\right)$$

where (Id + *θ*) is the diffeomorphic mapping of Ω_0_.

Then, the shape sensitivity can be defined by the derivative with respect to *θ*. The shape expression Equation (7) means that all admissible shapes will have the same topology as the initial shape Ω_0_. Therefore, the topology cannot be changed by continuously transforming the initial shape Ω_0_, which theoretically answers the reason why the level set topology optimization method cannot automatically nucleate holes.

Based on the above assumptions, the shape sensitivity of the objective function *J*(Ω) on Ω_0_ can be defined as the Fréchet derivative at *θ* = 0:

(9) $$J\left(\left(\mathrm{Id}+\theta \right)\left(\Omega_{0}\right)\right)=J\left(\Omega_{0}\right)+J^{\prime }\left(\Omega_{0}\right)\left(\theta \right)+ο\left(\theta \right),\;\mathrm{where}\;\underset{\theta \to 0}{\lim}\frac{\left|ο\left(\theta \right)\right|}{\left\|\theta \right\|}=0$$

where *J*(Ω) has first-order continuous differentiability at *θ* = 0. According to the definition of shape sensitivity, shape sensitivity Equation (9) is rewritten as:

(10) $$J^{\prime }\left(\Omega_{0}\right)\left(\theta \right)=\underset{\theta \to 0}{\lim}\frac{J\left(\left(\mathrm{Id}+\theta \right)\left(\Omega_{0}\right)\right)-J\left(\Omega_{0}\right)}{\theta }$$

Wang and Allaire have already calculated the shape sensitivity of the optimization problem Equation (3). This paper will directly quote the results, and the detailed process can refer to the work of Wang and Allaire [20,21].

**Lemma** **1.** *The shape sensitivity of objective compliance is:*

(11) $$\begin{array}{c} J^{\prime }\left(\Omega \right)\left(\theta \right)=\int_{\Gamma_{N}}\left(2\left[\frac{\partial \left(\tau \cdot u\right)}{\partial n}+H\tau \cdot u+Pu\right]-E\epsilon \left(u\right)\cdot \epsilon \left(u\right)\right)\theta \cdot nds\\ +\int_{\Gamma_{D}}\left(E\epsilon \left(u\right)\cdot \epsilon \left(u\right)\right)\theta \cdot nds\;\;\;\;\;\;\;\;\;\;\;\;\;\;\;\;\;\;\;\;\;\;\;\;\;\; \end{array}$$

**Lemma** **2.** *The shape sensitivity of the volume constraint is:*

(12) $$V^{\prime }\left(\Phi \right)\left(\theta \right)=\int_{\partial \Omega }\theta \left(x\right)\cdot n\left(x\right)ds$$

## 3. Bi-Directional Evolutionary Structural Optimization (BESO) and Topological Sensitivity

### 3.1. BESO

The topology optimization problem is often to maximize the stiffness (or minimize the compliance) of the structure under a given volume constraint. In BESO, the sensitivity number is used to remove low-efficiency materials or add high-efficiency materials. In other words, materials are removed from or added to the design domain by comparing the value of the sensitivity number. Therefore, BESO regards the structure itself as the design variable of the optimization problem. The optimization problem is expressed as:

(13) $$\begin{array}{c} \min\;\;C=F^{T}u=u^{T}Ku\;\;\;\;\;\;\;\;\;\\ \begin{array}{c} s.t.\;\;\;\sum_{i=1}^{N}V_{i}x_{i}\leq fV^{*}=V_{M}\\ F=Ku\;\;\;\;\;\;\;\;\;\;\;\;\;\;\;\;\; \end{array}\\ x_{i}=\{\begin{array}{c} 0\\ 1 \end{array}\;\;\;\;\;\;\;\;\;\;\;\;\;\;\;\;\;\; \end{array}$$

where compliance *C* is the objective of the optimization problem, ***F*** is the load vectors, ***u*** is the displacement vectors, ***K*** is the global stiffness matrix, *V_i_* is the elemental volume, V^*^ is the total volume of the design domain, *f* is the volume fraction, and *N* is the number of elements in the design domain, and the design variable *x_i_* is the elemental density with *x_i_* = 0 for a void element and *x_i_* = 1 for a solid element.

When a solid element is removed from the structure, the change of the mean compliance or total strain energy is equal to the elemental strain energy. This change is defined as the elemental sensitivity number:

(14) $$\alpha_{i}^{e}=\Delta C_{i}=u_{i}^{T}K_{i}u_{i}$$

When the structural meshes non-uniformly, the influence of the elemental volume needs to be considered, so the sensitivity number is changed to:

(15) $$\alpha_{i}^{e}=\frac{u_{i}^{T}K_{i}u_{i}}{V_{i}}$$

When the elements are void, the above sensitivity number is always 0, and no material can be added in the design domain. A sensitivity filter scheme is introduced to obtain the sensitivity number of the void elements. In addition, this filter method can solve the checkerboard and mesh dependency.

The formulation of sensitivity filter scheme is written as:

(16) $$\alpha_{i}=\frac{\sum_{j\in N_{i}}\omega \left(r_{ij}\right)\alpha_{j}^{n}}{\sum_{j\in N_{e}}\omega \left(r_{ij}\right)}$$

where *α_i_* is the elemental sensitivity after filtering, and $\alpha_{j}^{n}$ is the elemental sensitivity before filtering. The neighbor elements set *N_i_* of element *i* is defined as all elements whose spatial distance from the central cell *i* is less than or equal to the filtering radius *R_min_*. The weight factor *ω*(*r_ij_*) of the spatial distance is:

(17) $$\omega \left(r_{ij}\right)=R_{min}-r_{ij}$$

where *r_ij_* is the spatial distance between element *j* and central element *i*, defined as || *x_j_* − *x_i_* ||.

Although sensitivity filter can solve checkerboard and mesh dependency, the result is still a chaotic phenomenon, and the optimization fails to converge. The sensitivity number of the previous iteration is integrated into the sensitivity filter scheme to solve the instability phenomenon. In BESO, the average sensitivity number is frequently used to update the sensitivity, which is written as:

(18) $$\alpha_{i}=\frac{\alpha_{i}^{k}+\alpha_{i}^{k-1}}{2}$$

where *k* is the current iteration number.

Before adding materials to or removing materials from the current structure, the target volume *V^k^*^+1^ for the next iteration needs to be given. Since the volume constraint *V_M_* can be larger or smaller than the initial structure volume, the target volume in each iteration can be gradually reduced or increased until the constraint volume is reached. Therefore, the target volume can be expressed as:

(19) $$V^{k+1}=V^{k}\left(1\pm c_{er}\right)$$

where *c_er_* is the evolutionary volume ratio. If the current structure volume is equal to the volume constraint, the target volume *V^k^*^+1^ will remain *V_M_*.

After the above steps are completed, the structure can be updated according to the elemental sensitivity value *α_i_* after finite element analysis and sensitivity filtering. The elements are sorted according to the sensitivity number. The solid elements satisfying Equation (20) will be removed, and the void elements satisfying Equation (21) will be added.

(20) $$\alpha_{i}\leq \alpha_{rem}^{th}$$

(21) $$\alpha_{i}>\alpha_{add}^{th}$$

where $\alpha_{rem}^{th}\;$ and $\alpha_{add}^{th}$ are the sensitivity thresholds of removing and adding elements, respectively. Generally, let $\alpha_{rem}^{th}=\alpha_{add}^{th}=\alpha^{th}$, where $\alpha^{th}$ is determined by Equation (19). The detailed calculation of $\alpha^{th}$ can refer to Reference [10]. The finite element analysis, sensitivity filtering, and structure update are continuously looped until the volume constraint and the convergence criterion Equation (22) are satisfied.

(22) $$e=\frac{\left|\sum_{i}^{N}C_{k-i+1}-\sum_{i}^{N}C_{k-N-i+1}\right|}{\sum_{i}^{N}C_{k-i+1}}\leq \delta$$

where *e* is the change of objective, *δ* is the tolerance factor, and *N* is a positive integer.

### 3.2. Topological Sensitivity

**Figure 1:**
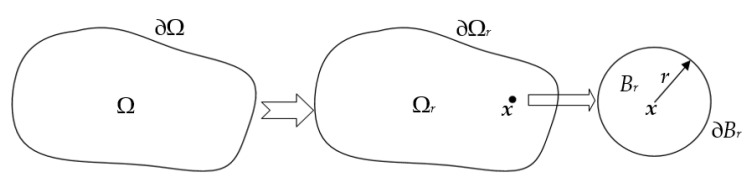
Initial definition of topological sensitivity.

The topological sensitivity defines the impact on the objective when a small hole is inserted at a certain position in the design domain. As shown in Figure 1, Ω∈ℝ^2^ is an open bounded domain, and its boundary ∂Ω is smooth enough, i.e., there is a normal vector **n** at an arbitrary point on the boundary. Inserting a small circular hole *B_r_* with a radius of *r* at the point ***x***∈Ω will produce a new bounded domain Ω*_r_* = Ω − *B_r_*, with a boundary of ∂Ω*_r_* = ∂Ω − ∂*B_r_*. Therefore, the topological sensitivity of the objective *J*(Ω) in a given design domain is defined as:

(23) $$D_{T}\left(x\right)=\underset{r\to 0}{lim}\frac{J\left(\Omega_{r}\right)-J\left(\Omega \right)}{M\left(r\right)}$$

where *M*(*r*) is the measure of the hole *B_r_*. According to reference [39], this paper takes the Lebesgue measure which can be written as:

(24) $$\underset{r\to 0}{lim}M\left(r\right)=0$$

As shown in Figure 1, a hole appears in the design domain Ω, which causes the topology changes of the design domain, i.e., Ω and Ω*_r_* are not homeomorphism. Therefore, topological sensitivity Equation (23) cannot be directly calculated.

**Figure 2:**
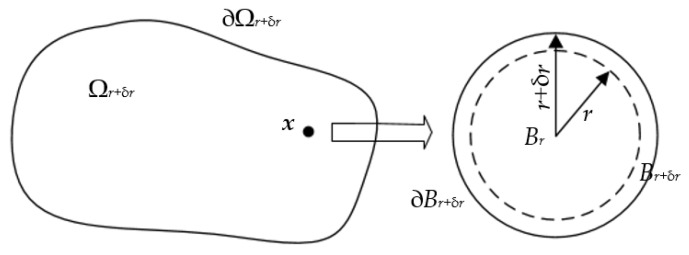
Modified definition of topological sensitivity.

To calculate the topological sensitivity, a new homeomorphic design domain needs to be reconstructed. According to the design domain Ω*_r_* with circular holes, a small variation δ*r* is employed to the initial radius *r*. The hole *B_r_* becomes a new hole *B_r_*_+δ*r*_ with a radius of *r*+δ*r*, so the design domain has also changed accordingly, from Ω*_r_* to Ω*_r_*_+δ*r*_ = Ω-*B_r_*_+δ*r*_, as shown in Figure 2. The modified topological sensitivity is defined as:

(25) $$D_{T}^{*}\left(x\right)=\underset{r\to 0}{lim}\left(\underset{\delta r\to 0}{lim}\frac{J\left(\Omega_{r+\delta r}\right)-J\left(\Omega_{r}\right)}{M\left(r+\delta r\right)-M\left(r\right)}\right)=\underset{\begin{array}{c} r\to 0\\ \delta r\to 0 \end{array}}{lim}\frac{J\left(\Omega_{r+\delta r}\right)-J\left(\Omega_{r}\right)}{M\left(r+\delta r\right)-M\left(r\right)}$$

According to the topological-shape sensitivity method [39], topological sensitivity can be solved by shape sensitivity. For the calculation of topological sensitivity, assume Ω*_r_*_+δ*r*_⇒Ω and Ω*_r_*⇒Ω_0_, then ∂Ω*_r_*_+δ*r*_⇒∂Ω and ∂Ω*_r_*⇒∂Ω_0_. Using the topology-shape sensitivity method, the relationship between shape sensitivity and topological sensitivity is expressed as:

(26) $$D_{T}\left(x\right)=D_{T}^{*}\left(x\right)=\underset{r\to 0}{lim}\frac{1}{M^{\prime }\left(r\right)\left|V_{N}\right|}{\frac{dJ\left(\Omega \right)}{d\theta }|}_{\theta =0}$$

This paper aims to study the topology optimization problem based on compliance, so it is necessary to solve the topological sensitivity of objective compliance. According to the research of Novotny et al. [36,37], the topological sensitivity of compliance is:

for *d* = 2
  (27) $$D_{T}J\left(x\right)=\frac{\pi \left(\lambda +2\mu \right)}{2\mu \left(\lambda +\mu \right)}\left(4\mu E\epsilon \left(u\right)\cdot \epsilon \left(u\right)+\left(\lambda -\mu \right)tr\left(E\epsilon \left(u\right)\right)tr\left(\epsilon \left(u\right)\right)\right)\left(x\right)$$
for *d* = 3
  (28) $$D_{T}J\left(x\right)=\frac{\pi \left(\lambda +2\mu \right)}{\mu \left(9\lambda +14\mu \right)}\left(20\mu E\epsilon \left(u\right)\cdot \epsilon \left(u\right)+\left(3\lambda -2\mu \right)tr\left(E\epsilon \left(u\right)\right)tr\left(\epsilon \left(u\right)\right)\right)\left(x\right)$$

where *λ* and *μ* are the Lamé moduli of the material, which satisfy:

(29) $$\lambda =\frac{E\nu }{1-\nu^{2}}$$

(30) $$\mu =\frac{E}{2\left(1+\nu \right)}$$

## 4. BESO Based on Topological Sensitivity

In this paper, CBT is used as a hole nucleation method for level set topology optimization. CBT replaces the sensitivity number in BESO with topological sensitivity. This section will describe the improved BESO method.

According to the definition of topological sensitivity, topological sensitivity is the impact of the objective with inserting a small hole in the design domain. BESO is to remove low-efficiency materials in the design domain based on a specific criterion. In other words, BESO can remove materials that have less impact on the objective, which is identical to the definition of topological sensitivity. This section combines BESO with topological sensitivity and uses topological sensitivity as the criterion for removing or adding materials to achieve the least impact on the objective, i.e., to achieve the optimal objective. Put simply, when the topological sensitivity value of a solid element is small, it means that removing the element has less impact on the objective. On the contrary, when the topological sensitivity value of void elements is large, it means that this position has a greater impact on the objective, so it is necessary to add materials here. Therefore, the sensitivity number *α_i_* of BESO can be replaced with the topological sensitivity:

(31) $$D_{T}^{i}=\underset{\epsilon \to 0}{lim}\frac{1}{M^{\prime }\left(\epsilon \right)\left|V_{N}\right|}{\frac{dJ\left(\Omega \right)}{d\theta }|}_{\theta =0}$$

According to the topological sensitivity of compliance, Equation (31) can be changed to:

for *d* = 2
  (32) $$D_{T}^{i}=\frac{\pi \left(\lambda +2\mu \right)}{2\mu \left(\lambda +\mu \right)}\left(4\mu E\epsilon \left(u\right)\cdot \epsilon \left(u\right)+\left(\lambda -\mu \right)tr\left(E\epsilon \left(u\right)\right)tr\left(\epsilon \left(u\right)\right)\right)\left(x\right)$$
for *d* = 3
  (33) $$D_{T}^{i}=\frac{\pi \left(\lambda +2\mu \right)}{\mu \left(9\lambda +14\mu \right)}\left(20\mu E\epsilon \left(u\right)\cdot \epsilon \left(u\right)+\left(3\lambda -2\mu \right)tr\left(E\epsilon \left(u\right)\right)tr\left(\epsilon \left(u\right)\right)\right)\left(x\right)$$

Correspondingly, to obtain the topological sensitivity of void elements while avoiding numerical instability, the same sensitivity filter method as traditional BESO is implemented, as shown in Equations (34) and (35). The subsequent target volume calculation and structure update are the same as the traditional BESO method, as shown in Equation (36), Equation (37) and Equation (38), respectively.

(34) $$D_{T}^{i}=\frac{\sum_{j\in N_{i}}\omega \left(r_{ij}\right)D_{T}^{j}}{\sum_{j\in N_{e}}\omega \left(r_{ij}\right)}$$

(35) $$D_{T}^{i}=\frac{{\left(D_{T}^{i}\right)}^{k}+{\left(D_{T}^{i}\right)}^{k+1}}{2}$$

(36) $$V^{k+1}=V^{k}\left(1\pm c_{er}\right)$$

(37) $$D_{T}^{i}\leq D_{T}{}_{rem}^{th}$$

(38) $$D_{T}^{i}>D_{T}{}_{add}^{th}$$

The optimization process of BESO based on topological sensitivity is as follows:

1. Discretize the design domain and initialize the structure;
2. Perform finite element analysis, and calculate the elemental topological sensitivity using Equation (31);
3. Filter topological sensitivity using Equations (34) and (35);
4. Calculate the target volume using Equation (36);
5. Update the structure using Equations (37) and (38);
6. Check whether the convergence condition is satisfied. If not, return to step 2 for a new iteration.

**Figure 3:**
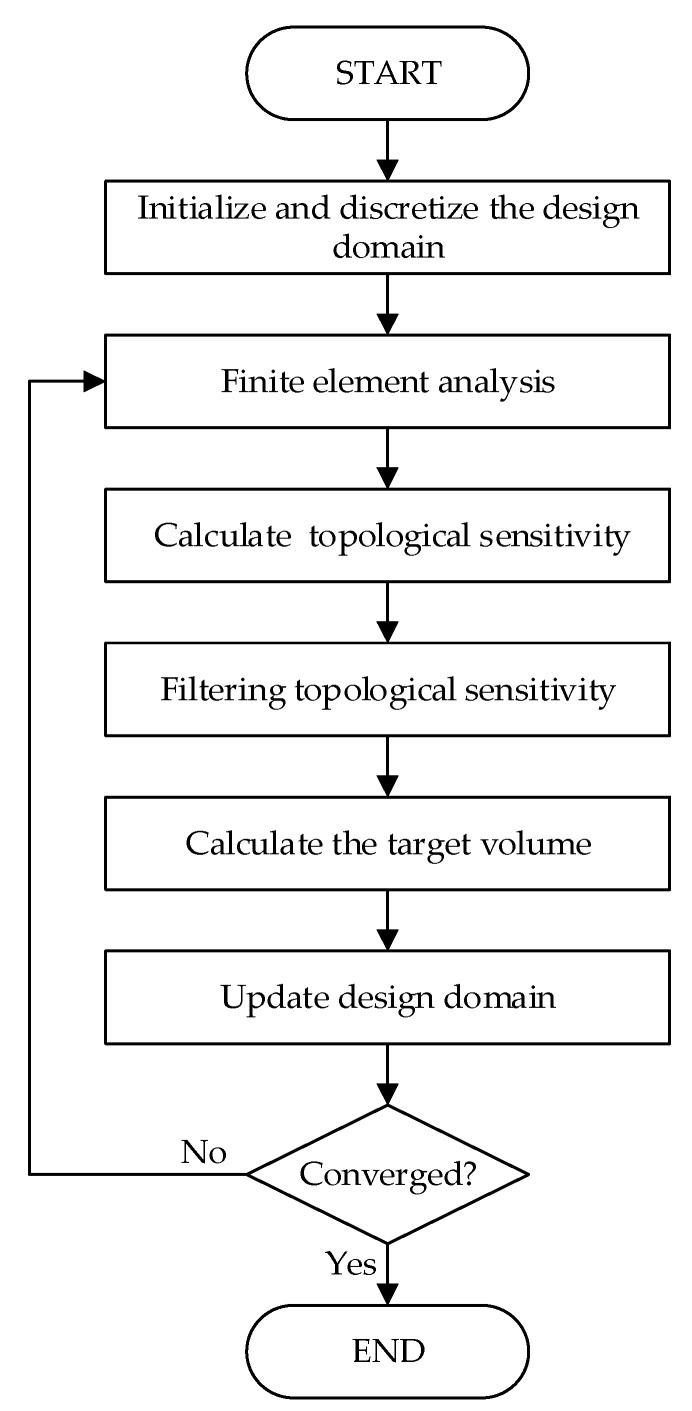
Flowchart of bi-directional evolutionary structural optimization (BESO) based on topological sensitivity.

The BESO optimization process based on topological sensitivity is shown in Figure 3.

To prove that topological sensitivity can be combined with BESO, a simple bridge beam and L-shaped beam are taken as numerical examples. When the optimization parameters and boundary conditions are the same, the traditional BESO and the improved BESO method are used to calculate the optimization problem, and the optimization results of the two methods are compared and analyzed.

**Example** **1.** *Simple bridge beam.*

**Figure 4:**
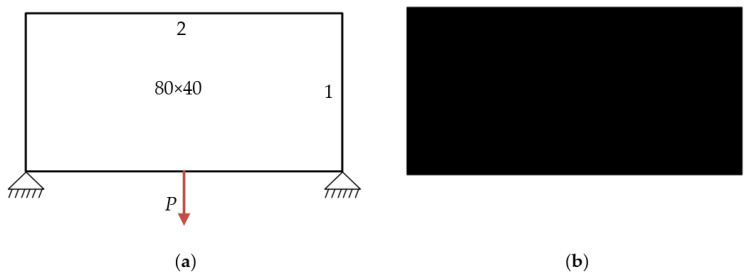
Optimal design problem of two-dimensional simple bridge beam, (**a**) design domain and boundary conditions; (**b**) initial shape.

**Figure 5:**
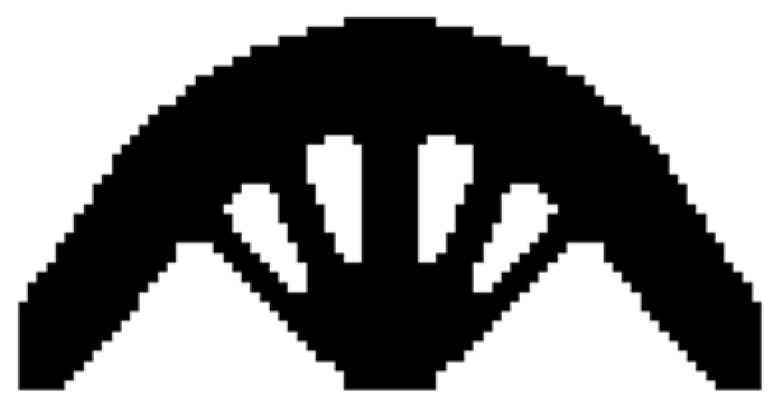
Optimized shape of simple bridge beam by traditional BESO.

**Figure 6:**
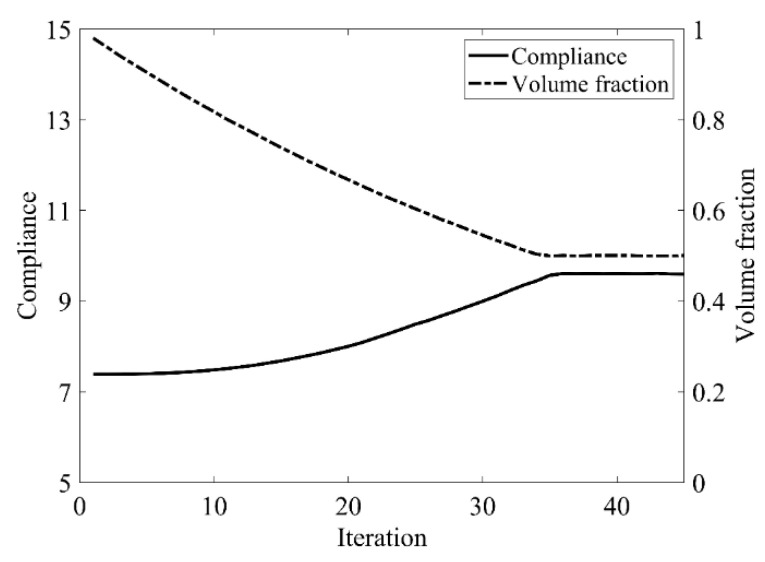
Convergence history of the compliance and volume fraction for two-dimensional simple bridge beam by traditional BESO.

The design domain of a simple bridge beam is a rectangular area with a ratio 2:1 of length *L* to height *H*, meshed with 80 × 40 = 3200 elements, as shown in Figure 4a. The shape is fixed at the bottom corners and a vertical load *P* = 1 is applied at the middle of the bottom side. In this setting, the Young’s modulus of the solid material is *E*_1_ = 1, the Young’s modulus of the void material is *E*_0_ = 10^−3^, and the Poisson’s ratio is *ϑ* = 0.3. The volume fraction upper *f* is limited to 0.5, the evolutionary volume ratio *c*_er_ is set to 0.02 and the filter radius *R_min_* is taken as 3. The initial shape is displayed in Figure 4b. Using traditional BESO to optimize the problem, the optimized shape is shown in Figure 5, and the evolution of the volume fraction and the compliance in the course of the optimization process are shown in Figure 6.

**Figure 7:**
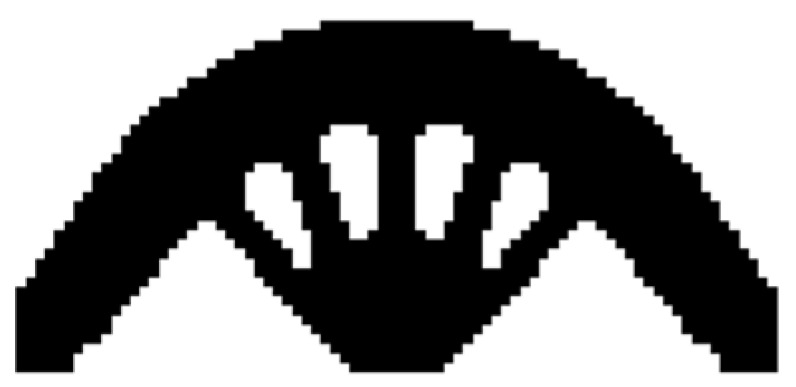
Optimized shape of simple bridge beam by the improved method.

**Figure 8:**
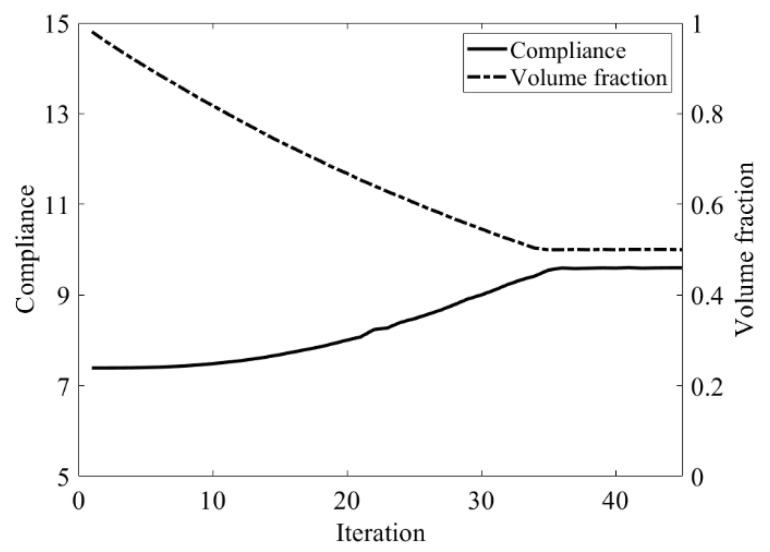
Convergence history of the compliance and volume fraction for two-dimensional simple bridge beam by the improved method.

Then using the improved method to optimize the problem, the optimized shape is shown in Figure 7, and the evolution of the volume fraction and the compliance in the course of the optimization process are shown in Figure 8.

Comparing the traditional BESO with the improved method, the topology of the final optimized shape is similar. As shown in Table 1, the compliance and the number of iterations are almost equal. Therefore, the topological sensitivity can be used to replace the sensitivity number in the traditional BESO for topology optimization.

**Example** **2.** *L-shaped beam.*

**Figure 9:**
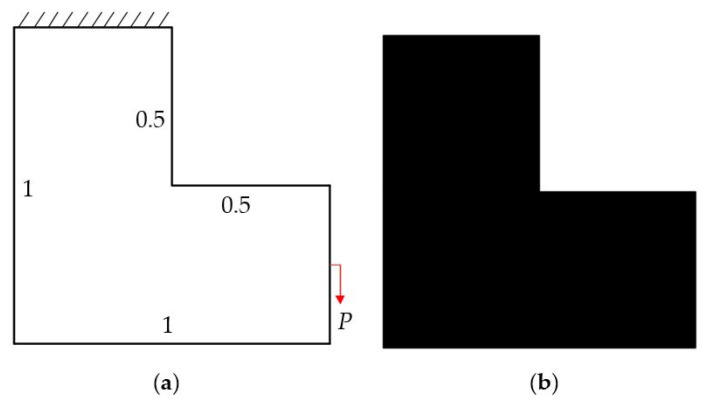
Optimal design problem of L-shaped beam, (**a**) design domain and boundary conditions; (**b**) initial shape.

**Figure 10:**
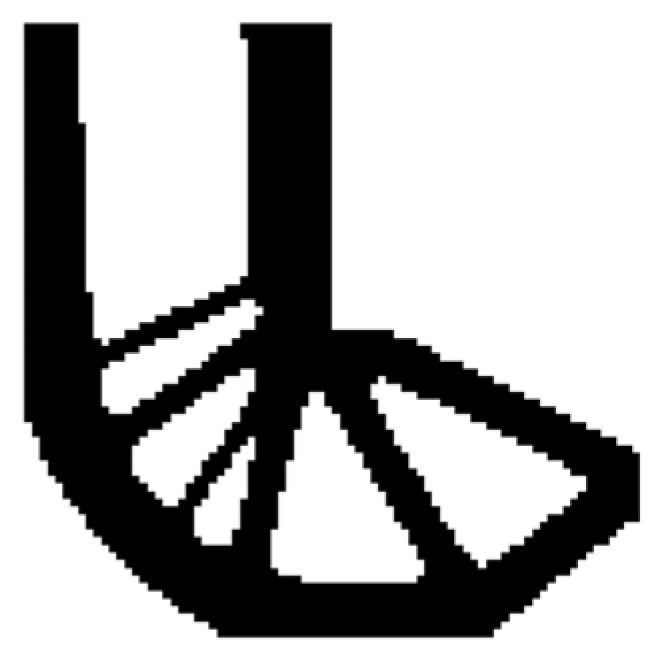
Optimized shape of L-shaped by traditional BESO.

**Figure 11:**
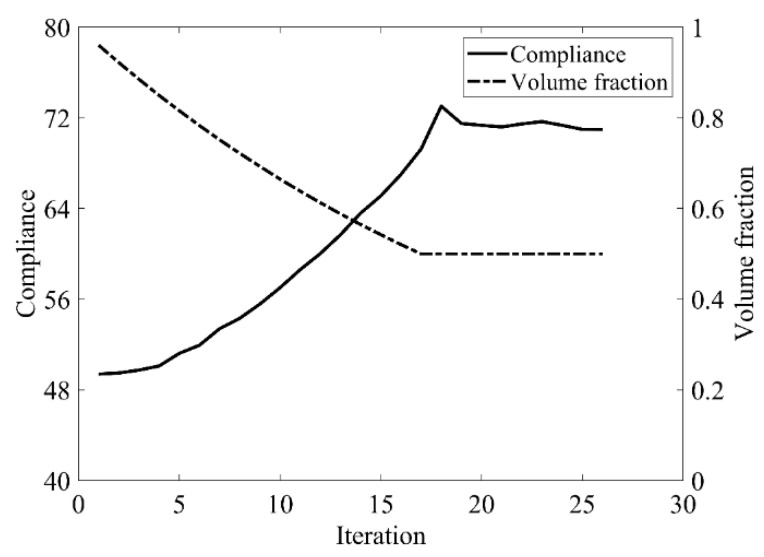
Convergence history of the compliance and the volume fraction for L-shaped beam by traditional BESO.

The design domain of this optimization problem is an L-shaped area with a ratio 1:1 of length *L* to height *H*, meshed with 4800 elements, as shown in Figure 9a. The shape is clamped at its upper side and a vertical load *P* = 1 is applied at the middle of its right-hand side. In this setting, the evolutionary volume ratio is *c_er_* = 0.04, and other optimization parameters are the same as those of the simple bridge beam. The initial shape is displayed in Figure 9b. Using traditional BESO to optimize the problem, the optimized shape is shown in Figure 10, and the evolution of the volume fraction and the compliance in the course of the optimization process are shown in Figure 11.

**Figure 12:**
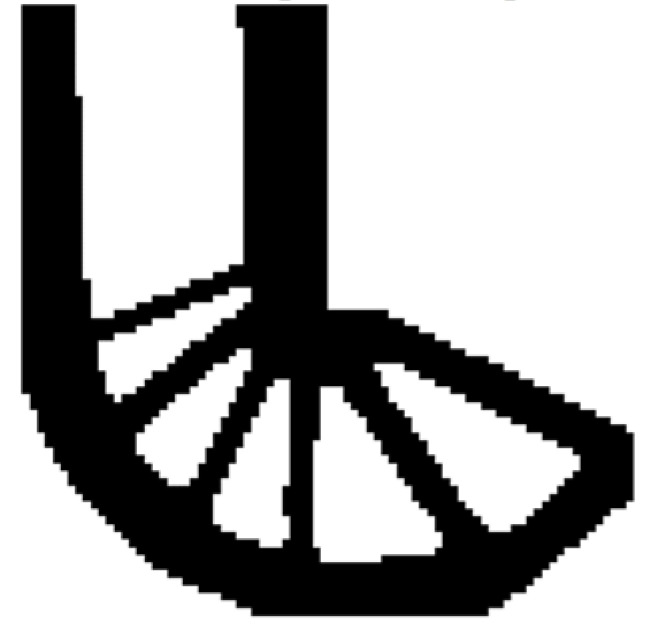
Optimized shape of L-shaped by the improved method.

**Figure 13:**
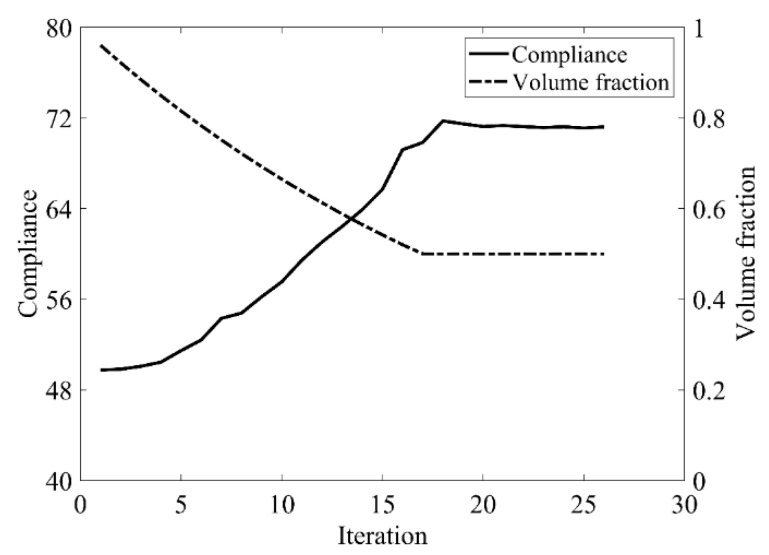
Convergence history of the compliance and the volume fraction for L-shaped beam by the improved method.

Then using the proposed method to optimize the problem, the optimized shape is shown in Figure 12, and the evolution of the volume fraction and the compliance in the course of the optimization process are shown in Figure 13.

Comparing the traditional BESO with the improved method, the topology of the final optimized shape is similar. Table 2 shows that the compliance is almost equal, so the topological sensitivity can be used to replace the sensitivity number in the traditional BESO for topology optimization.

The above two numerical examples prove that the method proposed in this paper can make the optimization problem converge to the optimal result. In other words, it is effective to use topological sensitivity to replace the sensitivity number in the traditional BESO method.

## 5. Combining BESO and Topological Sensitivity (CBT) Level Set Topology Optimization

In this paper, the Lagrangian method is used to solve the level set topology optimization problem Equation (3), which transforms the optimization problem into the Lagrangian unconstrained minimization problem. Therefore, the topology optimization problem can be rewritten as:

(39) $$\begin{array}{c} L\left(\Omega ,\ell ,\gamma \right)=J\left(\Omega \right)-\ell \left(V\left(\Phi \right)-V_{M}\right)+\frac{\gamma }{2}{\left(V\left(\Phi \right)-V_{M}\right)}^{2} \end{array}$$

where ℓ and *γ* are the Lagrangian multipliers and the penalty factors of the constraint function respectively, and their update rules are:

(40) $$\begin{array}{l} \ell^{n+1}=\ell^{n}-\gamma^{n}\left(V^{n}\left(\Phi \right)-V_{M}\right)\\ \gamma^{n+1}=\beta \gamma^{n} \end{array}$$

The shape sensitivity of the augmented Lagrangian function can be derived as:

(41) $$\begin{array}{c} L^{\prime }\left(\Omega ,\ell ,\gamma \right)\left(\theta \right)=J^{\prime }\left(\Omega \right)\left(\theta \right)-\ell \int_{\Omega }\theta \cdot nds+\gamma \left(V\left(\Phi \right)-V_{M}\right)\int_{\Omega }\theta \cdot nds \end{array}$$

Based on the shape sensitivity, the normal evolution velocity *V_n_* of the Hamilton–Jacobi equation can be obtained as:

(42) $$V_{n}=E\epsilon \left(u\right)\cdot \epsilon \left(u\right)-\ell +\gamma \left(V\left(\Phi \right)-V_{M}\right)$$

In Section 4, topology sensitivity is integrated into the BESO method, which is to prove that BESO can be combined with topology sensitivity. Next, CBT will be introduced to nucleate holes during the level set topology optimization. According to the idea of CBT, adding materials to and removing materials from the current structure requires the topological sensitivity threshold $D_{T}{}^{th}$. The threshold is usually determined according to a given evolutionary volume ratio. Assuming that there are N elements in the design domain, the topological sensitivity $D_{T}^{i}$ of all elements are sorted depending on the value, that is $D_{T}^{1}<D_{T}^{2}<\cdots <D_{T}^{V}<\cdots <D_{T}^{N}$. According to Equation (19), V elements are required to maintain holes (i.e., N-V solid elements), then the topological sensitivity threshold is:

(43) $$D_{T_{1}}^{th}=D_{T}^{V}$$

However, only using the threshold in Equation (43) is likely to cause unstable optimization. The BESO method will remove materials from the boundary of the structure when the structure is close to the optimization result. In this case, the boundary based on the evolution of the level set method will continue to be updated, and the material will be added at the position where the material was removed by the BESO method, which will easily cause numerical instability and fail to obtain the optimization result. Therefore, another topological sensitivity threshold $D_{T_{2}}^{th}$ needs to be introduced in the optimization, and its value is determined according to the average topological sensitivity of the structure boundary ∂Ω. The boundary threshold can be described as:

(44) $$D_{T_{2}}^{th}=\beta \bar{D_{T}}$$

where 0 < *β* < 1 is a user-defined threshold factor, and $\bar{D_{T}}$ is the average topological sensitivity of the structure boundary ∂Ω.

Therefore, the topological sensitivity threshold $D_{T}{}^{th}$ of adding and removing materials can be defined as:

(45) $$D_{T}{}^{th}=min(D_{T_{1}}^{th},D_{T_{2}}^{th})$$

The holes are nucleated every j iteration in the CBT method. After some iterations, if the topological sensitivity in the solid domain is greater than or equal to the threshold $D_{T}{}^{th}$, the hole nucleation process ends.

The detailed description of the CBT level set topology optimization procedure is as follows:

1. Define the design domain and initialize level set function;
2. Solve linear elasticity equation via the finite element method;
3. Calculate shape sensitivity, topological sensitivity and the normal evolution velocity $V_{n}$;
4. Solve the Hamilton–Jacobi equation to update the level set function;
5. If the current iteration number is an integer multiple of *j*, nucleate hole by Equation (45), then go to step 6. Otherwise, go to step 7;
6. Calculate the topological sensitivity threshold $D_{T}{}^{th}$;
7. Reinitialize the level set function;
8. Check whether the convergence criteria are satisfied. If not, repeat steps 2–8 until convergence.

**Figure 14:**
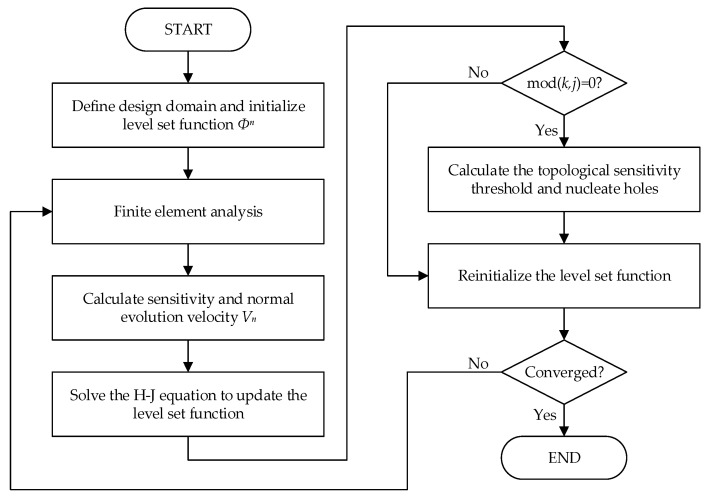
Flowchart of the combining BESO and topological sensitivity (CBT) level set topology optimization.

The flowchart of CBT level set topology optimization is shown in Figure 14.

## 6. Numerical Examples

In this section, three numerical examples are presented to validate the CBT method for nucleating holes. The objective of the optimization problem is to minimize the compliance of typical two-dimensional structures. In all cases, the material properties and loads are dimensionless. The materials for all numerical examples are isotropic. Assume that the Young’s modulus of the solid material is *E*_1_ = 1, the Young’s modulus of the void material is *E*_0_ = 10^−3^, and the Poisson’s ratio is *ϑ* = 0.3. All examples adopt four-node rectangular elements to mesh the design domain.

### 6.1. Simple Bridge Beam

**Figure 15:**
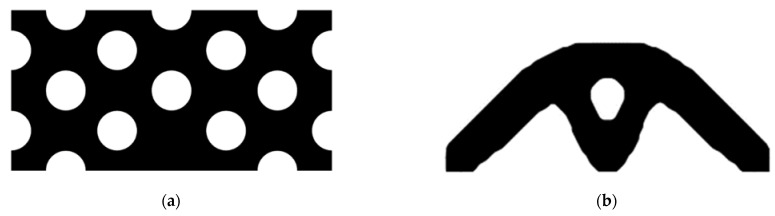
Optimal design problem of simple bridge beam by classical level set topology optimization, (**a**) initial shape; (**b**) optimized shape.

**Figure 16:**
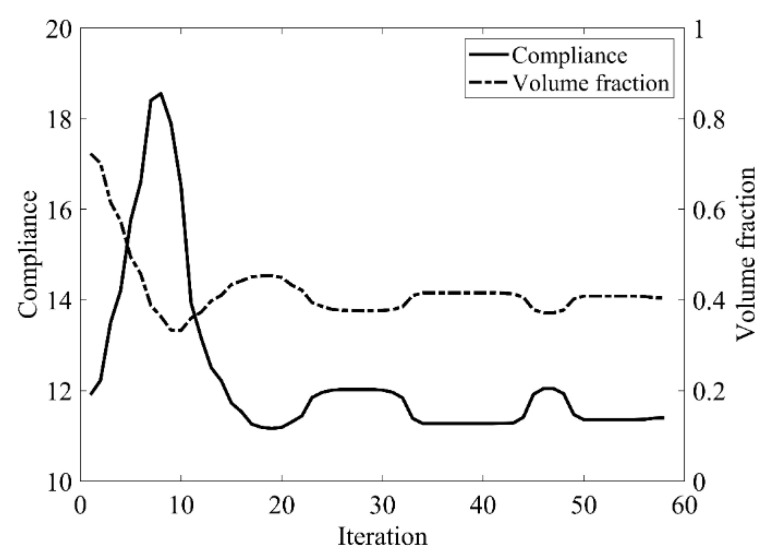
Convergence history of the compliance and the volume fraction for simple bridge beam by classical level set topology optimization.

The design domain of the simple bridge beam is meshed with 80 × 40 = 3200 elements, as shown in Figure 4a. In this setting, the volume fraction is *f* = 0.4. First, using the classical level set topology optimization to optimize the problem, the initial shape and the optimized shape are displayed in Figure 15, and the evolution of the volume fraction and the compliance in the optimization process are shown in Figure 16.

**Figure 17:**
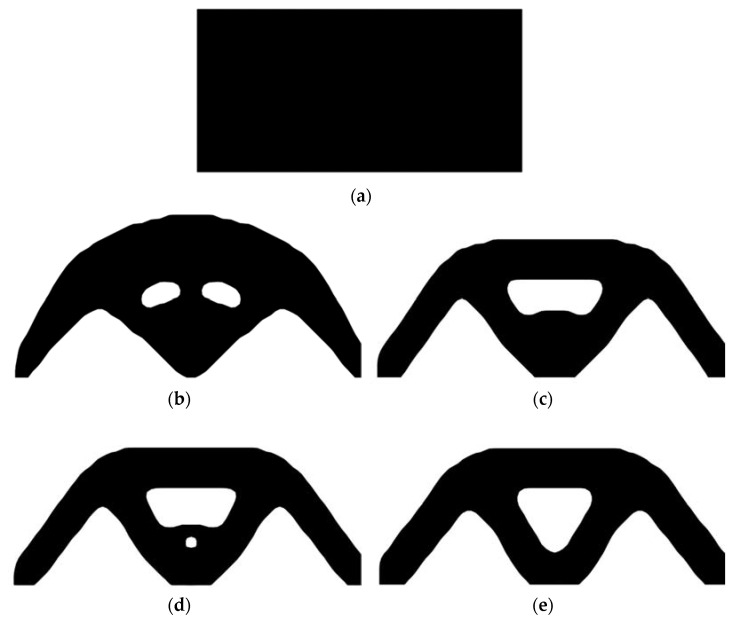
Optimal design problem of simple bridge beam by CBT level set topology optimization, (**a**) initial shape; (**b**) iteration 10; (**c**) iteration 20; (**d**) iteration 40; (**e**) optimized shape.

**Figure 18:**
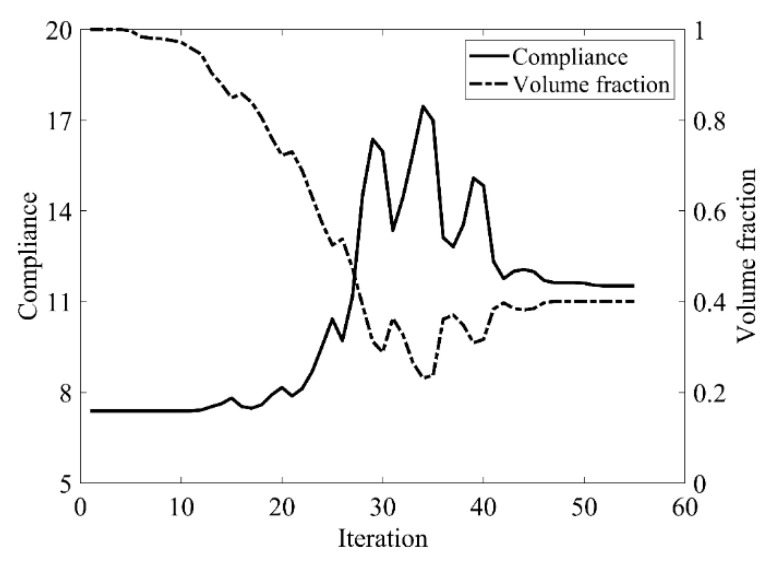
Convergence history of the compliance and the volume fraction for simple bridge beam by CBT level set topology optimization.

Second, let the evolutionary volume ratio is *c_er_* = 0.02, the filter radius *R_min_* = 3, and *j* = 5. Assume that the optimization problem is iterated from the full structure. The CBT level set method is used to optimize the problem. The optimization results are shown in Figure 17, where (a) is the initial shape, (b–d) are the intermediate results, and (e) is the final optimized shape. The evolution of the volume fraction and the compliance in the optimization process are shown in Figure 18.

Figure 18 shows that the compliance and volume fraction converge smoothly after 55 iterations. As shown in Table 3, the compliance and the number of iterations are almost equal to that of the classical level set method. Comparing the optimization results of the classical level set method and the CBT level set method, the topology of both is similar, while the initial structure of the CBT level set method is simpler, which is of great significance in practical engineering applications. For engineering designers, it is not necessary to guess the distribution of holes in the initial structure before optimization, which can reduce the design workload remarkably. This example proves that the CBT is effective in nucleating holes.

**Figure 19:**
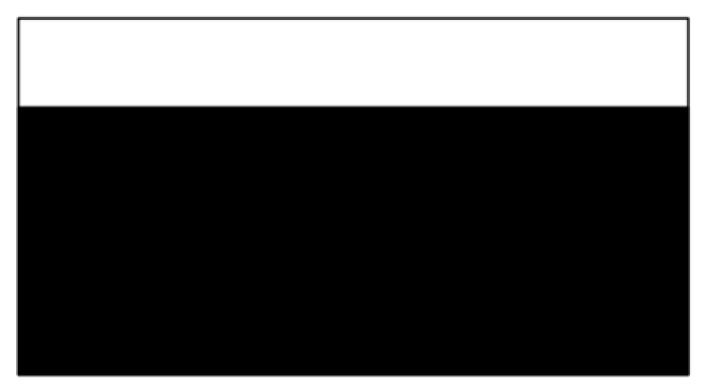
Optimal design problem of simple bridge beam with a volume fraction of 0.75.

**Figure 20:**
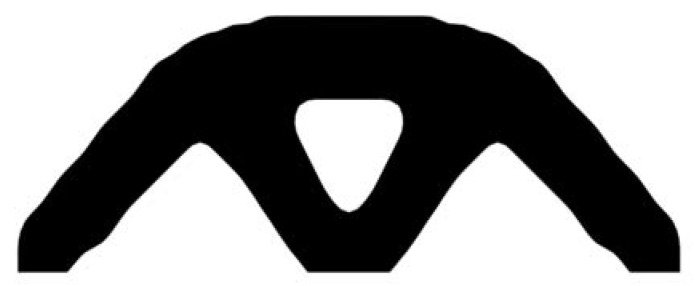
Optimized shape of simple bridge beam with a volume fraction of 0.75.

**Figure 21:**
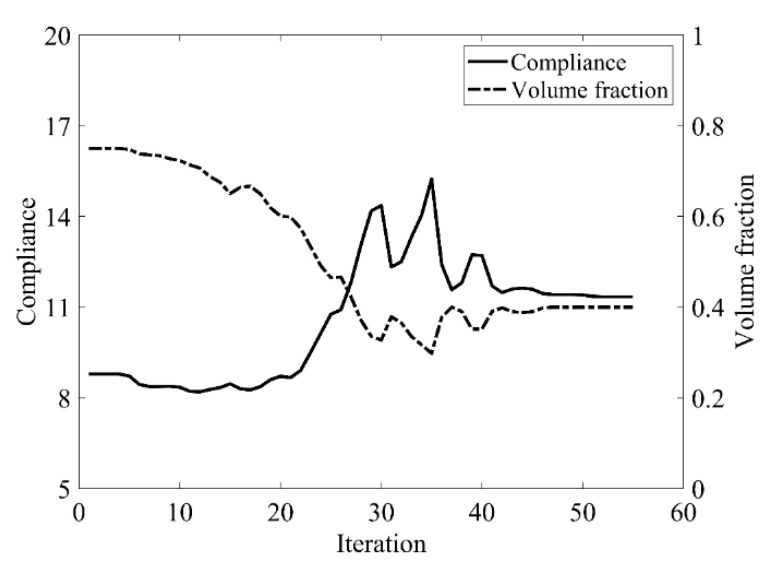
Convergence history of the compliance and the volume fraction for simple bridge beam by CBT level set topology optimization.

It is known that the BESO method can be optimized from an initial guess shape which has a volume equal or close to the objective volume. Next, an initial shape with a volume fraction of 0.75 will be optimized. The initial shape and the final optimized shape are shown in Figure 19 and Figure 20, respectively. The evolution of the volume fraction and the compliance in the optimization process are shown in Figure 21.

As shown in Figure 20, a similar optimization result is obtained when the initial volume fraction is 0.75. Although a structure with a smaller volume fraction can reduce the computational cost of finite element analysis, the number of iterations has not been reduced, causing the optimization problem to fail to converge. To make the optimization algorithm more robust, it is recommended to start optimization from the full structure.

### 6.2. Cantilever Beam

**Figure 22:**
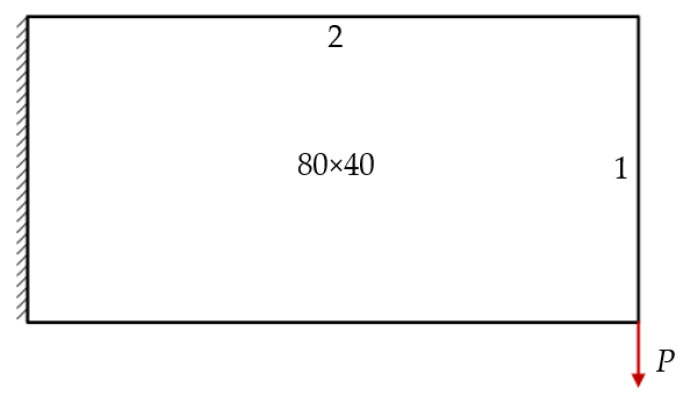
Design domain and boundary conditions of cantilever beam.

**Figure 23:**
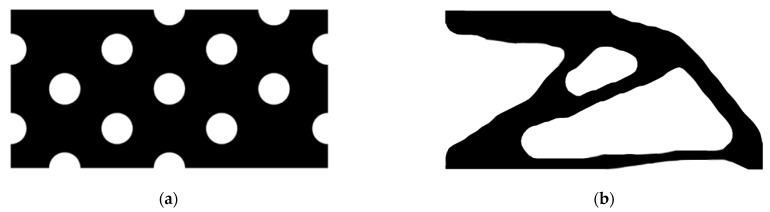
Optimal design problem of cantilever beam by classical level set topology optimization, (**a**) initial shape; (**b**) optimized shape.

**Figure 24:**
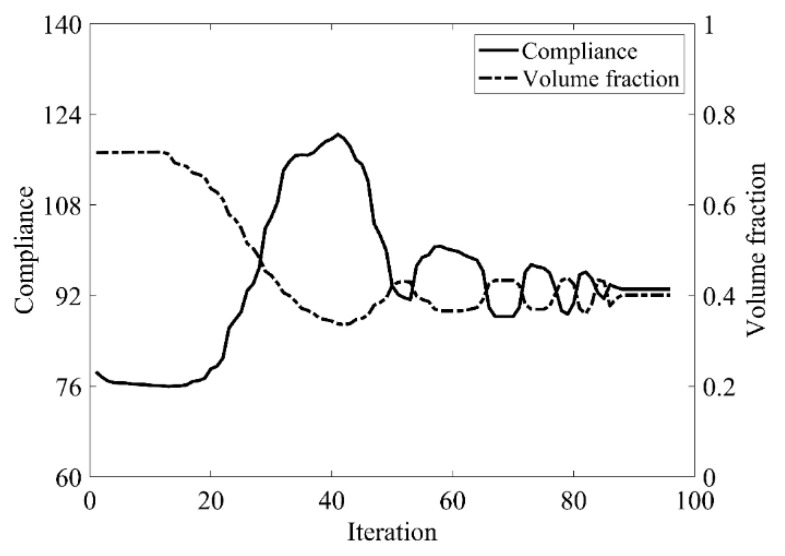
Convergence history of the compliance and the volume fraction for cantilever beam by classical level set topology optimization.

The design domain of the cantilever beam is a rectangular area with a ratio 2:1 of length *L* to height *H*, meshed with 80 × 40 = 3200 elements, as shown in Figure 22. The shape is fixed at its left-hand side and a vertical load *P* = 1 is applied at the bottom of its right-hand side. In this setting, the volume fraction is *f* = 0.4. First, using the classical level set topology optimization to optimize the problem, the initial shape and the optimized shape are displayed in Figure 23, and the evolution of the volume fraction and the compliance in the optimization process are shown in Figure 24.

**Figure 25:**
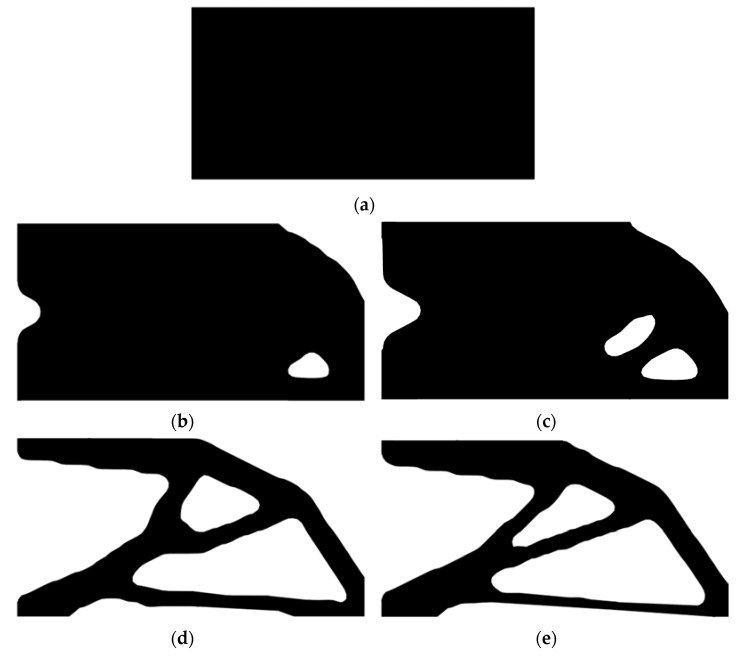
Optimal design problem of cantilever beam by CBT level set topology optimization, (**a**) initial shape; (**b**) iteration 20; (**c**) iteration 40; (**d**) iteration 60; (**e**) optimized shape.

**Figure 26:**
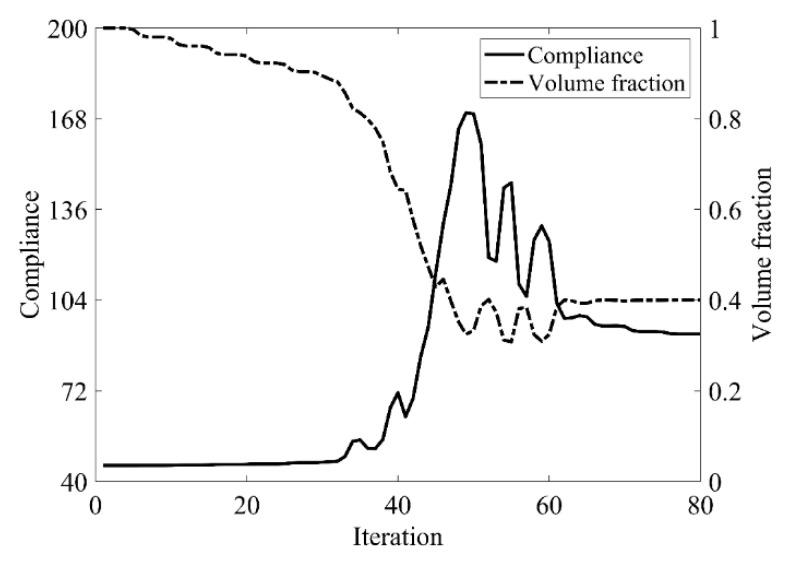
Convergence history of the compliance and the volume fraction for cantilever beam by CBT level set topology optimization.

Second, let the evolutionary volume ratio is *c_er_* = 0.02, the filter radius *R_min_* = 3, and *j* = 5. Assume that the optimization problem is iterated from the full structure. The CBT level set method is used to optimize the problem. The optimization results are shown in Figure 25, where (a) is the initial shape, (b–d) are the intermediate results, and (e) is the final optimized shape. The evolution of the volume fraction and the compliance in the optimization process are shown in Figure 26.

The cantilever beam topology optimization problem can converge smoothly from a full structure after 80 iterations. Comparing the traditional level set method with the CBT level set method, the topological shape of the optimized structure is similar. As shown in Table 4, the difference of compliance is small, and the number of iterations is lower than that of the classical level set method. The optimized results can confirm the validity and usefulness of CBT level set topology optimization.

### 6.3. L-Shaped Beam

**Figure 27:**
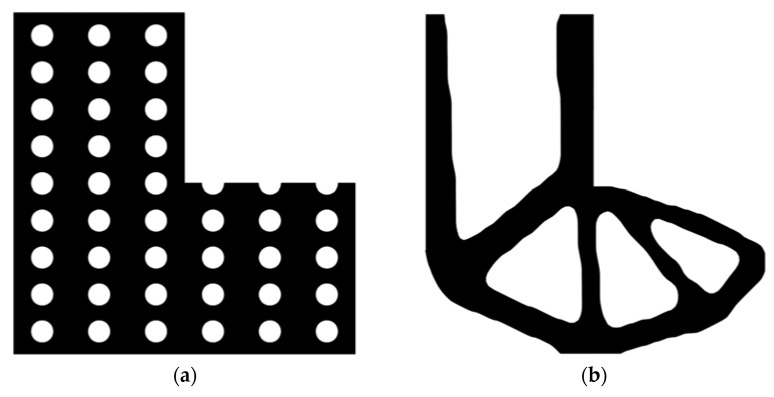
Optimal design problem of L-shaped beam by classical level set topology optimization, (**a**) initial shape; (**b**) optimized shape.

**Figure 28:**
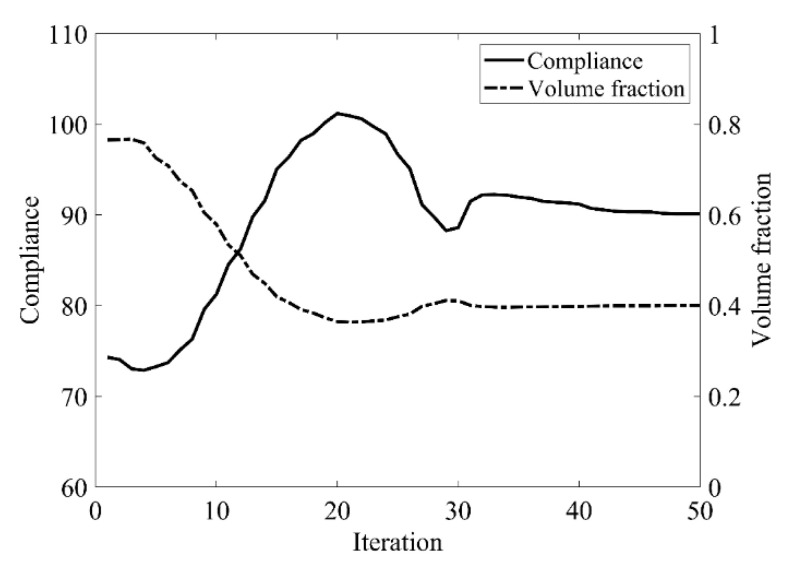
Convergence history of the compliance and the volume fraction for L-shaped beam by classical level set topology optimization.

The design domain of the L-shaped beam is meshed with 4800 elements, as shown in Figure 9a. In this setting, the volume fraction is *f* = 0.4. First, using the classical level set topology optimization to optimize the problem, the initial shape and the optimized shape are displayed in Figure 27, and the evolution of the volume fraction and the compliance in the optimization process are shown in Figure 28.

**Figure 29:**
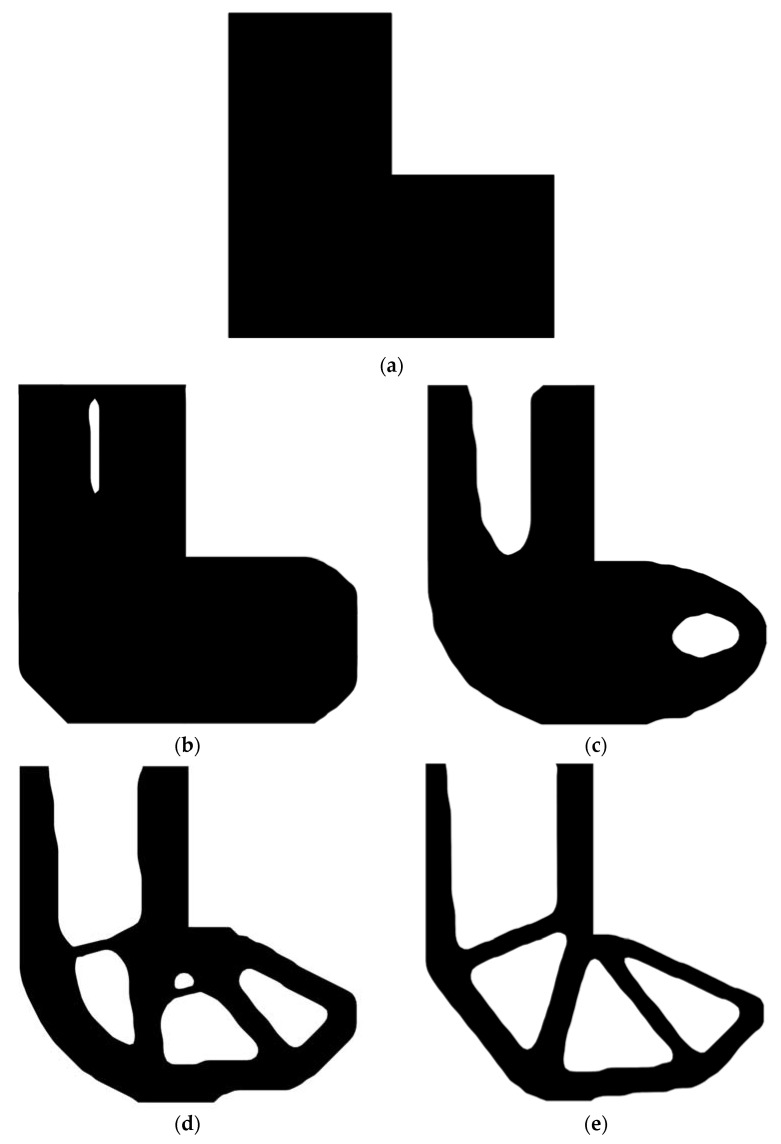
Optimal design problem of L-shaped beam by CBT level set topology optimization, (**a**)initial shape; (**b**) iteration; 10 (**c**) iteration 20; (**d**) iteration 30; (**e**) optimized shape.

**Figure 30:**
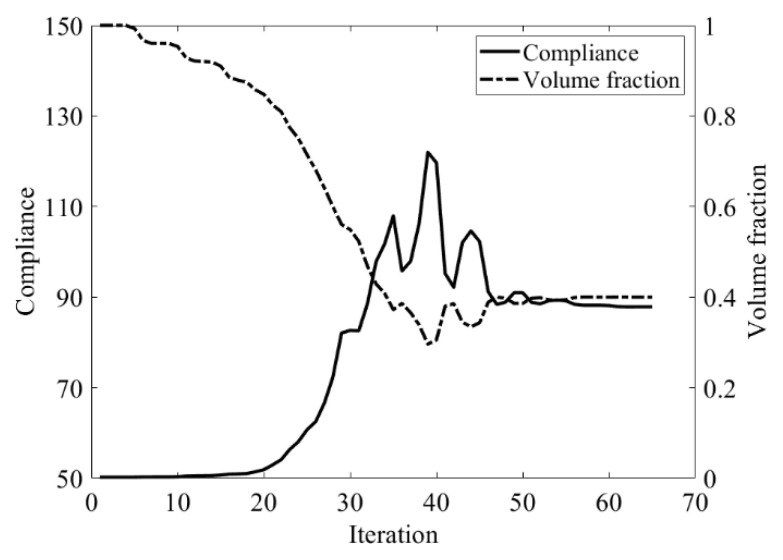
Convergence history of the compliance and the volume fraction for L-shaped beam by CBT level set topology optimization.

Second, let the evolutionary volume ratio is *c_er_* = 0.04, the filter radius *R_min_* = 3, and *j* = 5. Assume that the optimization problem is iterated from the full structure. The CBT level set method is used to optimize the problem. The optimization results are shown in Figure 29, where (a) is the initial shape, (b–d) are the intermediate results, and (e) is the final optimized shape. The evolution of the volume fraction and the compliance in the optimization process are shown in Figure 30.

The L-shaped beam topology optimization problem converges smoothly from a full structure after 65 iterations. Comparing the traditional level set method with the CBT level set method, the topological shape of the optimized structure is similar. As shown in Table 5, the difference of compliance is small, but the number of iterations increases. Since the initial shape of the classical level set method has more holes, it tends to the target volume fraction faster.

The above three numerical examples effectively prove that CBT topology optimization can iterate from the full initial structure to the optimal solution. Therefore, this method can be deemed to a new hole nucleation method and provides a new solution to the limitations of the classical level set method.

## 7. Conclusions

In this paper, the CBT level set topology optimization is proposed. The proposed method can achieve the automatic nucleation of holes in the optimization process. The optimization results of three two-dimensional numerical examples prove the effectiveness of the CBT method. In the CBT level set topology optimization, the sensitivity threshold $D_{T}^{V}$ is calculated by the evolutionary volume ratio and the boundary sensitivity threshold $\bar{D_{T}}$ is obtained to ensure the stability of the optimization. The CBT method is easy to program and, therefore, more acceptable to engineers. However, the analysis of topology sensitivity is complicated. Therefore, we do not claim that the proposed method is the best option for hole nucleation in all level set topology optimization problems. The proposed method should be considered as a good alternative to other successful hole nucleation methods. In future work, the CBT method can be applied to buckling problems or geometric non-linear problems and can be easily extended to multi-material topology optimization.

## Figures and Tables

**Table 1:** Results for topology optimization of the simple bridge beam.

	Traditional BESO	The Improved Method
Total element number	3200	3200
Volume fraction	0.5	0.5
Evolutionary volume ratio	0.02	0.02
Filter radius	3	3
Compliance	9.596	9.601
Total iteration	45	45

**Table 2:** Results for topology optimization of L-shaped beam.

	Traditional BESO	The Improved Method
Total element number	4800	4800
Volume fraction	0.5	0.5
Evolutionary volume ratio	0.04	0.04
Filter radius	3	3
Compliance	70.965	71.102
Total iteration	26	26

**Table 3:** Results for topology optimization of simple bridge beam.

	Classical Level Set Method	CBT Level Set Method
Total element number	3200	3200
Volume fraction	0.4	0.4
Evolutionary volume ratio	_	0.02
Filter radius	_	3
Compliance	11.399	11.526
Total iteration	58	55

**Table 4:** Results for topology optimization of cantilever beam.

	Classical Level Set Method	CBT Level Set Method
Total element number	3200	3200
Volume fraction	0.4	0.4
Evolutionary volume ratio	_	0.02
Filter radius	_	3
Compliance	93.150	92.064
Total iteration	96	80

**Table 5:** Results for topology optimization of L-shaped beam.

	Classical Level Set Method	CBT Level Set Method
Total element number	4800	4800
Volume fraction	0.4	0.4
Evolutionary volume ratio	_	0.04
Filter radius	_	3
Compliance	90.126	87.835
Total iteration	50	65

## Data Availability

Data available on request. The data presented in this study are available on request from the corresponding author.
